# Do patients think cannabis causes schizophrenia? - A qualitative study on the causal beliefs of cannabis using patients with schizophrenia

**DOI:** 10.1186/1477-7517-7-22

**Published:** 2010-09-28

**Authors:** Anna Buadze, Rudolf Stohler, Beate Schulze, Michael Schaub, Michael Liebrenz

**Affiliations:** 1 Psychiatric University Hospital, Research Group on Substance Use Disorders, Selnaustrasse 9, 8001 Zurich, Switzerland; 2 University of Zurich, Center for Disaster and Military Psychiatry, Zurich, Switzerland and University of Leipzig, Department of Social Medicine, Leipzig, Germany; 3 Research Institute for Public Health and Addiction, Zurich, Switzerland

## Abstract

**Background:**

There has been a considerable amount of debate among the research community whether cannabis use may cause schizophrenia and whether cannabis use of patients with schizophrenia is associated with earlier and more frequent relapses. Considering that studies exploring patients' view on controversial topics have contributed to our understanding of important clinical issues, it is surprising how little these views have been explored to add to our understanding of the link between cannabis and psychosis. The present study was designed to elucidate whether patients with schizophrenia who use cannabis believe that its use has caused their schizophrenia and to explore these patients other beliefs and perceptions about the effects of the drug.

**Methods:**

We recruited ten consecutive patients fulfilling criteria for paranoid schizophrenia and for a harmful use of/dependence from cannabis (ICD-10 F20.0 + F12.1 or F12.2) from the in- and outpatient clinic of the Psychiatric University Hospital Zurich. They were interviewed using qualitative methodology. Furthermore, information on amount, frequency, and effects of use was obtained. A grounded theory approach to data analysis was taken to evaluate findings.

**Results:**

None of the patients described a causal link between the use of cannabis and their schizophrenia. Disease models included upbringing under difficult circumstances (5) or use of substances other than cannabis (e. g. hallucinogens, 3). Two patients gave other reasons. Four patients considered cannabis a therapeutic aid and reported that positive effects (reduction of anxiety and tension) prevailed over its possible disadvantages (exacerbation of positive symptoms).

**Conclusions:**

Patients with schizophrenia did not establish a causal link between schizophrenia and the use of cannabis. We suggest that clinicians consider our findings in their work with patients suffering from these co-occurring disorders. Withholding treatment or excluding patients from certain treatment settings like day-care facilities or in patient care because of their use of cannabis, may cause additional harm to this already heavily burdened patient group.

## Background

There still is a debate among the research community whether cannabis use may cause schizophrenia [1,2] and whether cannabis use of patients with schizophrenia might lead to a more untoward outcome like earlier and more frequent relapses [3].

Arguments in this debate primarily stem from cohort studies [4], systematic reviews [5], and meta analyses [6]. Considering that studies exploring patients' view on a controversial topic have contributed to our knowledge of important clinical issues [7], such as patients' reasons for following or refusing medical recommendations [8,9], and patients needs and wishes at the end of life [10], it is surprising how little these views have been explored to add to our understanding of the link between cannabis and psychosis.

Even though patients' beliefs on the role of cannabis in the pathogenesis of schizophrenia have - to our knowledge - not been studied so far, some studies have explored reasons for cannabis and/or other substance use in psychosis, mainly by means of questionnaires (e.g. the Reasons for Cannabis Use Questionnaire, the Psychosis and Drug Abuse Scale (PADAS) and the Cannabis Use Effects Survey [11-13]).

Generally, it was found that the most frequent reasons for cannabis use among individuals with psychotic disorders were similar to those of healthy subjects (e. g. a wish to relax, to be high, to reduce boredom, social motives [e. g. "to go along with the group"], improving sleep, anxiety, and agitation). However, some of these studies also found that an important part of patients (11-40%) reported to use cannabis to reduce hallucinations [12,14,15].

These rather unexpected statements stand in contradiction to the widely held belief of most clinicians that cannabis use is specifically unhealthy for patients with schizophrenia and underline the importance of integrating patients' views into treatment, since they may influence adherence to medical treatment and therefore have an impact on the overall outcome [8,16].

The present exploratory study uses a grounded theory approach, conducting narrative interviews [17] to explore - to our knowledge for the first time - disease models of patients with schizophrenia who use cannabis and to clarify whether patients believe in a causal link between cannabis use and schizophrenia.

## Method

Ten consecutive patients fulfilling criteria for schizophrenia (ICD-10 F20.0) and for a current harmful use of/dependence from cannabis (ICD-10 F12.1 or F12.2) were recruited from the in- and outpatient clinic of the Psychiatric University Hospital Zurich. Further inclusion criteria included being 18 years of age or older and willingness to give written informed consent. Exclusion criteria were insufficient language skills, acute psychosis or intoxication and a diagnosis of personality disorder. For each patient the full patient's chart from the clinic with a complete biographical and psychiatric history was available, including diagnosis according to ICD-10.

In order to identify patients' personal perceptions and motivations we used single, unstructured, in depth interviews [18] lasting for about 1.5 hours. Our topic guide (*vide infra*) provided a flexible interview framework. A definitive version of it had emerged after the 4th narrative. Interviews started with a narrative opening question concerning patient's subjective disease models. Probes were used to explore the role of cannabis in case it was not covered spontaneously in patients' initial narrative. In addition, we allowed themes and motives identified in earlier interviews to be explored in those following, combining the principles of maximum variation and complexity reduction to simultaneously widen the scope of results and examining previous assumptions [19].

Care was taken to shape a conversation in which the patient felt free to present his or her own view [20]. All interviews were conducted by the same researcher in the outpatient clinic. Patients received a compensation of 20 Swiss Francs for participation. In addition to the interviews, information on prescribed medication and exact diagnosis (ICD-10) was obtained from clinical records.

A grounded theory approach to data analysis was taken to evaluate findings. This meant allowing the data to "speak for themselves" rather than approaching the data within existing theoretical frameworks [21]. All interviews were tape recorded and then transcribed in full. Transcripts were compared with tapes by the research team and validated with patients, if necessary. Validation of transcripts with patients was necessary in three cases. This was due to technical difficulty (e.g. distracting side noise on tape).

Materials were coded using an inductive qualitative procedure [22]. Categories obtained were discussed in the research team to validate ratings and achieve consensus. AB applied the final code, with confirmation of consistency through blind dual coding of two transcripts with ML. Authorization by the local ethics committee was obtained before the study was conducted. All patients were assured complete confidentiality and provided their written informed consent to the study, specifically to the tape-recorded interviews.

### Topic Guide

« How would you describe the condition you are suffering from? »

« What is/are the cause/s of your illness? »

« You might have heard that there might be a relationship between the consumption of cannabis and the likelihood of developing a mental disorder at a later time. How do you feel about that? »

« What are the effects if you consume cannabis? »

« Do these effects vary over time or condition you are in when smoking? »

## Results

Patients' characteristics, diagnosis and consumption patterns are described in table 1.

**Table 1:** Patients' characteristics, cannabis use, and medication

subject	age	sex	Initials	use of cannabis					prescribed drugs at time of interview**	diagnosis (ICD-10)	occupation
				**starting age**	**years of consumption**	**max. quanity in joints/d**	**way of consumption**	**frequency of use**			

**VP1**	26	m	P.O.	12	14	10	smoking	2-3/w	none	F20.0 F12.24 F20.0,	disability pension commercial clerk,
**VP2**	27	m	P.A.	16	11	7	smoking	2/w	C, AD, B	F11.22, F12.25, F14.26, F13.20	disability pension medical school
**VP3**	42	m	M.B.	14/15	27	12	smoking	daily	N	F20.0 F.12.25	drop out commercial clerk,
**VP4**	27	m	K.C.	12	15	20	smoking	2/w	M, B, N	F20.0, F11.22, F12.24	disability pension commercial clerk,
**VP5**	36	m	S.F.	16	20	3	smoking	daily	N, AD	F20.0, F12.25, F33.0	50% workload lumberjack,
**VP6**	43	m	H.G	20	23	15	smoking	1-2/w	N, AD	F11.22, F12.24, F20.0	disability pension high school drop
**VP7**	34	m	Z.A.	17	17	15	smoking	sporadic	N, MS, M	F20.03, F12.20	out, disability pension
**VP8**	51	m	R.S.	17	34	10	smoking	sporadic	N, MS, AD	F20.0, F43.1, F14.2, F12.24	mason, disability pension
**VP9**	44	f	N.A.	20	24	5	smoking	sporadic	N	F41.3, F20.0,12.24	social worker, 100% workload
**VP10**	53	f	B.L.	16	37	4	smoking	sporadic	N, B	F20.01, F11.22, F12.2, F50.1	waitress, 100% workload

**AD = antidepressants; N = neuroleptics; R = Ritalin^®^; C = Concerta^®^; B = benzodiazepines; M = modafinil; MS = mood stabilizers

### Patients' explanatory models about the origin of their mental illness

Nine of the interviewed patients regarded themselves as suffering from a mental disorder. While seven patients identified themselves as suffering from schizophrenia, one viewed himself as being depressed and one described himself as emotionally unstable. One patient did not regard himself as suffering from a mental disorder at all, but stated to only have some mental health problems.

In our sample all patients had developed thorough explanatory models about the origin of their mental condition. Although each individual etiological concept was unique in its own way, it was possible to identify common major themes and shared features. It has to be noted, however, that some overlapping between themes occurred. Three of the ten patients identified more than one contributing cause and had adopted a multi-factorial explanatory model.

We identified five major themes on perceived causation, which we characterize below, starting with the most commonly expressed perception.

#### Family induced

Most frequently patients attributed the development of their mental disorder to their upbringing under difficult circumstances, comprising parental neglect, parental overprotection, and physical or psychological abuse:

«... I suffer from paranoia. That is because I was beaten at home and I was neglected. What is left over is anxiety. It is still enough if someone raises the voice to me. It will scare me. Both my parents, my father and my mother, beat me physically...»

S.F. 36y. male, cannabis use since age 16

«...It all started when I was around 12 - 13 years old. My parents were constantly quarrelling, so I began daydreaming: Everything what happened was just a theatrical play with me becoming merely a facade. Because of that I had no chance to relax. The lack of relaxation is the reason, why I suffer from schizophrenia today...»

M.B. 40y. male, cannabis use since age 14

«...Discrimination. My mother completely denied me when she got a new partner. I got my voices because of the denial. The entire story with my psychosis started because of discrimination...»

N.A. 44y. female, cannabis use since age 20

#### Drug induced

Repeatedly patients described an alteration of their mental state, after a moderate or excessive use of one or more legal or illegal substances. They thought that this alteration had led to a permanent damage of their mental health. Our patients attributed the capability of inducing such changes solely to hallucinogens and non-hallucinogenic amphetamines. Interestingly, cannabis was rarely mentioned in this context.

«...To put it simply: Excessive labor of the brain, because of to much methamphetamine. That was difficult. I went to a friend of mine, banged against her door and told her that she should be watching out for me... It went on for an entire night, it just was too much. I think the reason (for my illness) is an overwork of my brain...»

K.C. 27y. male, cannabis use since age 12

«...I once took too much ecstasy. They took me to the Klinik Hard (regional hospital in the Zurich area) and after this event it was a gradual process until I got schizophrenia...»

P.O. 26y. male, cannabis use since age 12

«...I had used other stuff before, LSD, so I had heard voices at the age of 17-18 years. Also alcohol played a major role in developing this illness...»

H.G. 43y. male, cannabis use since age 20

«...At a party I was finally offered a drug cocktail containing stimulants and LSD and that is when my illness really got rolling...»

Z.A. 34y. male, cannabis use since age 17

#### Socially induced

Some explanatory models given by patients focused on social factors as the main reason for developing schizophrenia. Factors like loneliness, conflicts in partnership, and problems at work or school were mentioned, but were not seen as causally related. The main motive identified was "social pressure" as exemplified below:

«...My family put a lot of social pressure on me and I began to feel stressed. My siblings are all very successful and have prestigious jobs. My sister is an attorney, my older brother is a doctor, and the other is a philologist. During my first years of attending school, I was successful as well. But then I started to feel a lot of pressure and a lot of stress. I am sure that my illness was to some extent induced by that stress...»

Z.A. 34y. male, cannabis use since age 17

#### "Biological" or genetic models

Two patients put forward biological explanations for why they were suffering from schizophrenia. Interestingly though, biological explanations were only given as part of a multi-factorial approach, when patients described more than one contributing cause for their illness.

«...On the other hand I am burdened by a family history with schizophrenia and that has practically to do with it as well. My grandfather had this disposition...»

Z.A. 34y. male, cannabis use since age 17

«...All three girls have problems. One of my sisters is in psychiatric treatment as well. One person was contagious, like spreading influenza... this person was my mother»

N.A. 44y. female, cannabis use since age 20

#### Esoteric models

Magical explanatory models were adopted by two of our patients. It has to be noted that both patients had an immigrant background.

«...Having fever is being sick. Having cancer is being sick. Vomiting is being sick. Having a headache is being sick. What I have... I always have the feeling that I do not have everything under control. But it is not really an illness. It is power, and god gives this power to me. I can hear my mother and my father and also other dead people. I have been off to war and I have killed people. People came to me. And now I am scared of those people. I can see through people. One glimpse is enough. This is a gift by god...»

R.S. 51y. male, cannabis use since age 17

«...Back then I was very interested in occultism and that is why I later got into trouble. I started to hear voices and I was followed by demons...»

P.A. 27y. male, cannabis use since age 16

### Patients' view of a causal relationship between cannabis use and schizophrenia

As seen above, one of the explanatory models was connected to the use of substances, mainly focusing on the role of hallucinogens and amphetamines. However, none of the participants made a direct link between cannabis consumption and the onset of psychotic symptoms in their initial narrative statements. This was surprising to us since a potential causal relationship between cannabis use and schizophrenia is currently widely discussed in the Swiss public. Upon further exploration concerning the effects of their cannabis consumption, patients expressed very differentiated views on that topic.

First, patients presupposed **a clear temporal order **between consuming cannabis and the occurrence of psychotic symptoms for assigning a causal role to their substance use:

«...I have tried to answer this question myself. I really tried to put both things into connection after I had a "mega"-psychosis. But I can assure you, it came not because of cannabis, this is not the origin of it. I mean I believe that it can make some things more apparent. Not must, but can. Really, it cannot be the origin of it all, because I had these feelings, long before I started with it...»

*M.B*.

In this context, cannabis use was clearly framed as a consequence of psychotic symptoms rather than their origin:

«...The scenario is this: You first go crazy and then you start to smoke. It's not the origin... it is rather that you try to medicate yourself. ...»

*Z.A*.

Further, those questioned reasoned that possible negative effects of cannabis are related to the **quality of the cannabis preparation**. Differentiating between "good" and "bad" cannabis, they associated "good" cannabis with positive effects, while the use of "bad" cannabis was seen as resulting in negative experiences. In other words, they feel on the safe side as long as they assure using high quality preparations:

«...There is no causal relationship. Some slight influence: It depends on the quality of it. Sometimes it's worse, sometimes it's better, but most often you feel better...»

*S.F*.

In addition, patients stated that, in their view, **adverse reactions to cannabis are dose-related:**

«...I am mentally unstable, not very stable. It can really put me into it (psychosis) if I smoke every day...once, or twice per week that is fine, at the maximum three times. You know, one glass of red wine per day is fine too... as long as it does not become three bottles. It all depends on the amount. For me cannabis is a medical plant. You really can grow old with it. Not like with hard drugs...»

*P.O*.

«...No. I would say if you consume it within a normal range it could have positive results. If you overdo it then results will follow. If you take it together with cocaine symptoms just intensify. I got really anxious and then I started hearing voices...»

*P.A*.

Study participants moreover believed that the effects of cannabis are **varying between different individuals**. In these contexts, patients might use downward social comparisons with sufferers experiencing more severe psychotic symptoms than themselves by considering the latter to be "more vulnerable" and thus feel somewhat protected themselves:

«...Cannabis might have played a minor role. I just believe that cannabis is different in every person. You cannot really generalize it. ...»

*K.C*.

Finally, some study participants **denied a causal connection **between cannabis use and their illness **altogether**. This may be motivated by the fact that they clearly distinguish between the causation of their illness and an exacerbation of individual symptoms. As the following statements suggest:

«...It did not cause the voices...(but) it disturbs my memory when I use it a lot...»

*H.G*.

«...No. I do not see a connection between my mental problems and cannabis...»

*N.A*.

### Effects of cannabis use, its advantages and disadvantages

Our study patients regarded the use of cannabis as an important way of self-regulation and self-medication.

While most patients had a rather positive view of cannabis and used it (specifically) to reduce tension, to attenuate symptoms of depression or (simply) for relaxation purposes, some raised concerns especially because of its worsening psychotic symptoms and anxiety. Other drawbacks of using cannabis included its causing of increasing feelings of indifference and its acute aggravating of cognitive deficits.

Frequently it was a shared belief among all patients that positive aspects prevailed over possible disadvantages of cannabis use.

Most frequently, those questioned described that cannabis served them **to reduce anxiety and tension **Patients' statements may point to the fact they patients perceive cannabis particularly helpful in reducing positive psychotic symptoms:

«...When I smoke it I am better, I am less scared and I don't have bad dreams anymore. I can "regenerate my feelings" with it. Cannabis is an aid. It takes the speed out of my thoughts, I can see my own thoughts and I can arrange them properly...»

*P.O*.

«...I can calm down using it. It has something of a ritual too, when I come home after work at night. It can reduce my anxiety. However, if it is bad stuff, it can aggravate anxiety too. I just can lead a better life with cannabis. It makes my problems bearable...»

*S.F*.

«...The voices (with my schizophrenia it is like this - I only hear words, not sentences but words) go away. It calms me down. It also reduces my chronic pain and it loosens me up...»

*N.A*.

«... In the beginning I was fascinated by it, because I could relax so easily. It helped me to put all other things aside...»

*P.A*.

One patient even envisages a role of cannabis in suicide prevention, assuming that stopping to use it may exacerbate symptoms to such an extent that suicidal behavior might ensue:

«...During psychosis it was different. I felt that something was going on and I thought that cannabis might have different effects altogether. So I stopped using it during this time. I had not used it for days when I jumped...» (The patient had committed a suicide attempt, by jumping out of the window)

M.B

Patients further identified benefits of cannabis in alleviating blunted affect, social withdrawal and lack of motivation. Here, participants ascribed **energizing and mood-lifting effects **to their cannabis use:

«...I see clear advantages. I am more of a "lonesome wolf". When my spirits are low, it is even worse. I don't want to see anybody. A joint can reverse this. Before I go to work I need to smoke in order to reduce my inner tension somewhat. It disturbs my memory when I use it a lot.

Sometimes when I go shopping and I return, I have forgotten something that I knew I wanted to get before...»

*H.G*.

«...You cannot even imagine how tired I was. With it I was more awake and I could think clearly. My tiredness was attenuated and I felt more composed. In the beginning it even improved my concentration and my memory. Disadvantages: If it is of bad quality it makes me feel indifferent...»

*Z.A*.

«...I have more fantasy and I am more creative. In school it really helped. I could write better essays. I could read more and I was more active. With cannabis I can also bear my crazy thoughts...disconnect. ...»

«...I use cannabis as an antidepressant medication...»

*M.B*.

Some participants expressed a **differentiated view on the effects of cannabis**. In their view, effects of consumption differ between phases of the illness and their personal state, also implying negative outcomes if using cannabis in the "wrong circumstances":

«...In 90% of the times I don't have adverse effects. In 10% of the times I get flashbacks. That is a price I am willing to pay... Cannabis enhances my feelings either way. If I have a bad day I will become more depressed, on a good day I become more joyful...»

*P.O*.

«... When I first started using it and I was really nervous and I smoked marijuana I was panicking... »

*R.S*.

## Discussion

In this present exploratory study we examined disease models expressed by cannabis using patients with schizophrenia and clarified whether this patient group suspected a causal link between cannabis use and their illness.

We identified five major motives in the disease models of schizophrenia patients with a co-occurring abuse of cannabis: Mental illness was attributed towards upbringing under difficult familial circumstances, to social pressure, and to the use of legal and illegal substances. Additionally, genetic and esoteric explanations were given.

These motives do not fundamentally differ from explanatory models that have been elucidated in a study on schizophrenia patients without a co-occurring substance use disorder. Using a semi-structured interview, Angermeyer et al. (1988) identified recent psychosocial factors, personality, family, biology, and esoteric reasons as explanations put forward by patients suffering from schizophrenia, schizo-affective disorders, and "affective psychosis" [23].

Three patients favored a multi-modal explanation, identifying more than one reason as causes for their disorder. Again, this finding is in line with the results of previous quantitative [23] and semi-quantitative studies [24]. Their causal explanations further reflect the stress-vulnerability model underlying recent studies on the etiology of psychosis in a framework of gene-environment interaction [25]. However, patients tended to prefer psychological and social causes over genetic explanations for their condition regardless of whether they thought they suffered from schizophrenia or from other disturbances.

Interestingly, in this sample patients made a direct connection between the use of hallucinogens and amphetamines and the development of a mental illness at a later time. With regard to cannabis such a relationship was not made. One the contrary, apart from some adverse effects, (e.g. induction of flashbacks and perturbance of memory) patients had a rather positive view of cannabis and stated to use it to reduce tension, to attenuate symptoms of depression, to blunt the disturbing effects of acoustic hallucinations, or for relaxation purposes.

Furthermore, patients repeatedly described that they had experienced mental health problems well before they had started using cannabis, thus negating a causal relationship between the use of cannabis and the onset of schizophrenia all together.

Previous studies had found that reasons for cannabis use among individuals with psychotic disorders mainly comprised boredom, social motives, the wish to improve sleep, anxiety and agitation, control of negative and positive psychotic symptoms, increased energy levels, and improved cognitive function [12,13].

A recent review, categorized reasons for cannabis use in 4 main groups: enhancement of positive feelings, relief of dysphoria, social reasons, and reasons related to the illness and side effects of medication. It was found that patients most commonly describe enhancement of positive affect, relief from dysphoria and social enhancement. Fewer patients reported reasons related to relief of psychotic symptoms or relief of side effects of medication. Patients sometimes stated that cannabis negatively affected positive symptoms [26].

Our results are in accordance with these findings.

The differentiation between "good" and "bad" cannabis might relate to the findings of variable potency of cannabis extracts and its different ratios of Δ9-THC, CBD, and other pharmacological active ingredients [27]. However, it has also been known for a long time that the effects of cannabis are socially "constructed" and may vary across different situations and expectations [28].

Our study has some important limitations. First, this is an exploratory study aiming at an in-depth understanding of patients' views, thus using a small sample. Second, all study patients were recruited from the same treatment facility. Thus, it is unclear to what extent the present findings can be generalized. The present results should be verified in further studies involving more and more diverse patients.

## Conclusion

In summary, we found that patients with schizophrenia had a rather positive view of cannabis. This perception was reflected in their individual models of illness, which negated a causal link between schizophrenia and cannabis. Thus, we suggest that clinicians consider these findings in their work with patients suffering from such co-occurring disorders. Frequently, cannabis use of patients with schizophrenia has only been seen as a misbehavior leading to an untoward treatment outcome. Therefore, it often resulted and still results in withholding treatment or excluding such patients from certain treatment settings like day-care facilities or fulltime hospitals (RS, personal communication). In view of the beliefs surrounding cannabis use described above the majority of this patient group may not understand such an intervention. Rather patients may construe this therapeutic strategy as social rejection on the part of their therapists, which has been shown to significantly contribute to experiences of stigma and discrimination [29,30]. Stigma, then, has been found to act as an environmental risk factor for the onset and course of schizophrenia [31]. Further, treatment approaches which do not take account of patients' subjective illness models are not likely to enhance early help-seeking for psychosis and treatment collaboration [32]. Moreover, given the missing scientific evidence of deleterious effects of cannabis use on the course of schizophrenia [3], we think that such confrontational approaches cause additional harm to this already heavily burdened patient group. In conclusion patients' causal attributions and individual recovery strategies should be routinely explored in the context of the doctor-patient-relationship.

## Competing interests

The authors declare that they have no competing interests.

## Authors' contributions

AB, ML, RS, BS contributed to the design and the coordination of the study. All authors helped to draft the manuscript. All authors read and approved the final version of the manuscript.
